# Case Report: Exceptional response to perioperative pembrolizumab in UV-signature melanoma of unknown primary presenting as an isolated pulmonary metastasis

**DOI:** 10.3389/fonc.2026.1860040

**Published:** 2026-09-08

**Authors:** Austin Drysch, Genti Gjyzeli, Paul Buttars, Sunandana Chandra, Kalvin Lung

**Affiliations:** 1 Division of Thoracic Surgery, Northwestern University Feinberg School of Medicine, Chicago, IL, United States; 2 Department of Pathology, Northwestern University Feinberg School of Medicine, Chicago, IL, United States; 3 Department of Pathology & Immunology, Washington University School of Medicine, St. Louis, MO, United States; 4 Division of Hematology and Oncology, Northwestern University Feinberg School of Medicine, Chicago, IL, United States

**Keywords:** complete pathologic response, immune checkpoint inhibitors, melanoma of unknown primary, perioperative pembrolizumab, tumor mutational burden, ultraviolet signature

## Abstract

Melanoma of unknown primary (MUP) rarely presents as an isolated pulmonary mass, and the role of neoadjuvant immunotherapy in this setting remains undefined. We report a 26-year-old peripartum woman with systemic lupus erythematosus (SLE) and Sjögren’s syndrome who presented with a 12-cm centrally invasive pulmonary melanoma encasing the right pulmonary artery. Endobronchial biopsy confirmed melanoma, and genomic profiling demonstrated a very high tumor mutational burden (TMB; 39.2 mutations per megabase) and ultraviolet (UV) mutational signature, a profile most consistent with MUP. Given the extent of vascular involvement, the operative morbidity of upfront resection, and her autoimmune disease, single-agent neoadjuvant pembrolizumab (200 mg intravenously every 3 weeks) was initiated to facilitate resection while limiting the risk of immune-related toxicity. After three cycles, imaging demonstrated marked tumor regression, and the patient underwent right pneumonectomy with pulmonary artery reconstruction. Pathology demonstrated a complete pathologic response with no viable tumor and negative margins. She completed 15 adjuvant cycles, and surveillance imaging has shown no evidence of recurrence. To our knowledge, this is among the first reported cases of neoadjuvant PD-1 blockade enabling resection of MUP presenting as a solitary pulmonary mass. This case illustrates how immunogenomic features may inform therapeutic sequencing and how immunotherapy can support surgical decision-making in complex thoracic malignancies.

## Introduction

Melanoma is an aggressive neoplasm with high metastatic potential and significant mortality risk in advanced stages. Its global incidence continues to rise, with approximately 325,000 new cases reported in 2020, the majority attributable to ultraviolet (UV) radiation exposure (1). While programmed death-1 (PD-1) inhibitors have transformed outcomes for patients with advanced and resectable melanoma, durable responses remain variable, and PD-L1 expression alone is insufficient as a predictive biomarker (2–4). Increasing evidence suggests that tumor mutational processes, particularly UV radiation-associated mutagenesis, play a critical role in shaping tumor immunogenicity, neoantigen burden, and responsiveness to immunotherapy (5).

Melanoma of unknown primary (MUP) represents a distinct clinical and biologic subset, accounting for approximately 2-5% of melanoma cases. These tumors lack an identifiable cutaneous, mucosal, or ocular primary lesion and are hypothesized to arise from immune-mediated regression of the primary tumor (6–8). Molecular studies have demonstrated that MUP frequently harbors UV mutational signatures and canonical melanoma driver alterations—most commonly *BRAF*, *NRAS*, and *TERT*-promoter mutations—supporting a cutaneous origin despite the absence of a detectable primary lesion (9–11). This immune-selective biology has been proposed as a potential explanation for the favorable outcomes observed in some MUP cohorts and raises the possibility that MUP represents a particularly immunotherapy-responsive subtype (12).

Solitary pulmonary melanoma presents an additional diagnostic challenge. Although melanoma of the lung is formally recognized by the World Health Organization as a distinct diagnosis, its existence as a discrete clinicopathologic entity remains controversial (13–15). Contemporary molecular analyses have demonstrated that most “lung-only” melanomas exhibit UV signature mutations and oncogenic alterations characteristic of cutaneous melanoma, supporting the assessment that these lesions represent metastases from regressed or clinically occult primaries (15–18).

We present a case of melanoma of unknown primary presenting as a large, isolated pulmonary metastasis in a young postpartum patient who achieved a complete pathologic response (pCR) following perioperative pembrolizumab. Although this case met diagnostic criteria for melanoma of the lung, the molecular and clinical findings were most consistent with MUP presenting as an isolated pulmonary metastasis. This case is notable for its exceptional response to PD-1 blockade in the setting of a UV signature and a mutational profile with *NRAS*, *TERT*-promoter, and *PTEN* alterations. The presentation in the late peripartum period and the need for pulmonary artery resection and reconstruction on cardiopulmonary bypass further underscore the complex clinical context.

## Case report

A 26-year-old G1P0 woman with history of systemic lupus erythematosus (SLE), Sjögren’s syndrome, and scleroderma presented to Northwestern Medicine (NM) for high-risk delivery. She had initially sought care at an outside hospital for a persistent three-month cough. Chest computed tomography (CT) revealed a 12-cm mediastinal mass invading the right pulmonary artery with near-complete occlusion and encirclement of the right mainstem bronchus. Due to hypoxia requiring up to 10L of supplemental oxygen and fetal bradycardia with prolonged decelerations, she was transferred to NM. On arrival she was found to have severe preeclampsia requiring emergency caesarean delivery the following morning at 36 weeks and 6 days, resulting in the birth of a healthy boy.

**Figure 1:**
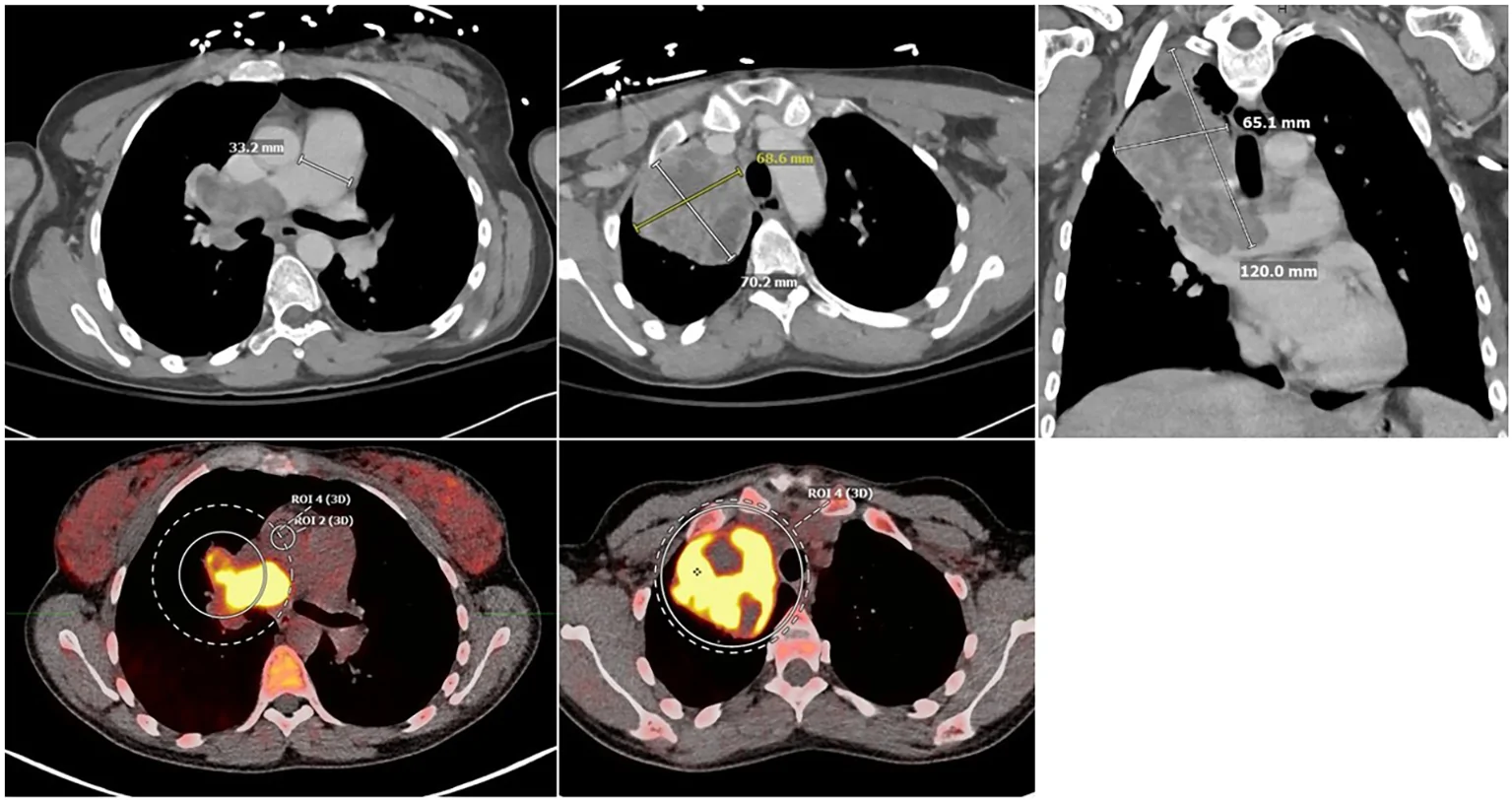
CT and PET-CT performed before pembrolizumab treatment, demonstrating maximal dimensions of the lesion and pulmonary artery involvement.

After delivery, she was weaned from supplemental oxygen and remained stable on room air. Preeclampsia was managed with nifedipine, diuretics, and labetalol. Two days post-delivery, PET-CT confirmed a 12-cm hypermetabolic mass in the right mediastinum without distant metastases or nodal involvement (**Figure 1**). Endobronchial ultrasound-guided transbronchial biopsy was performed the same day. The patient was subsequently discharged after a 5-day hospitalization with follow-up scheduled in medical oncology and thoracic surgery.

**Figure 2:**
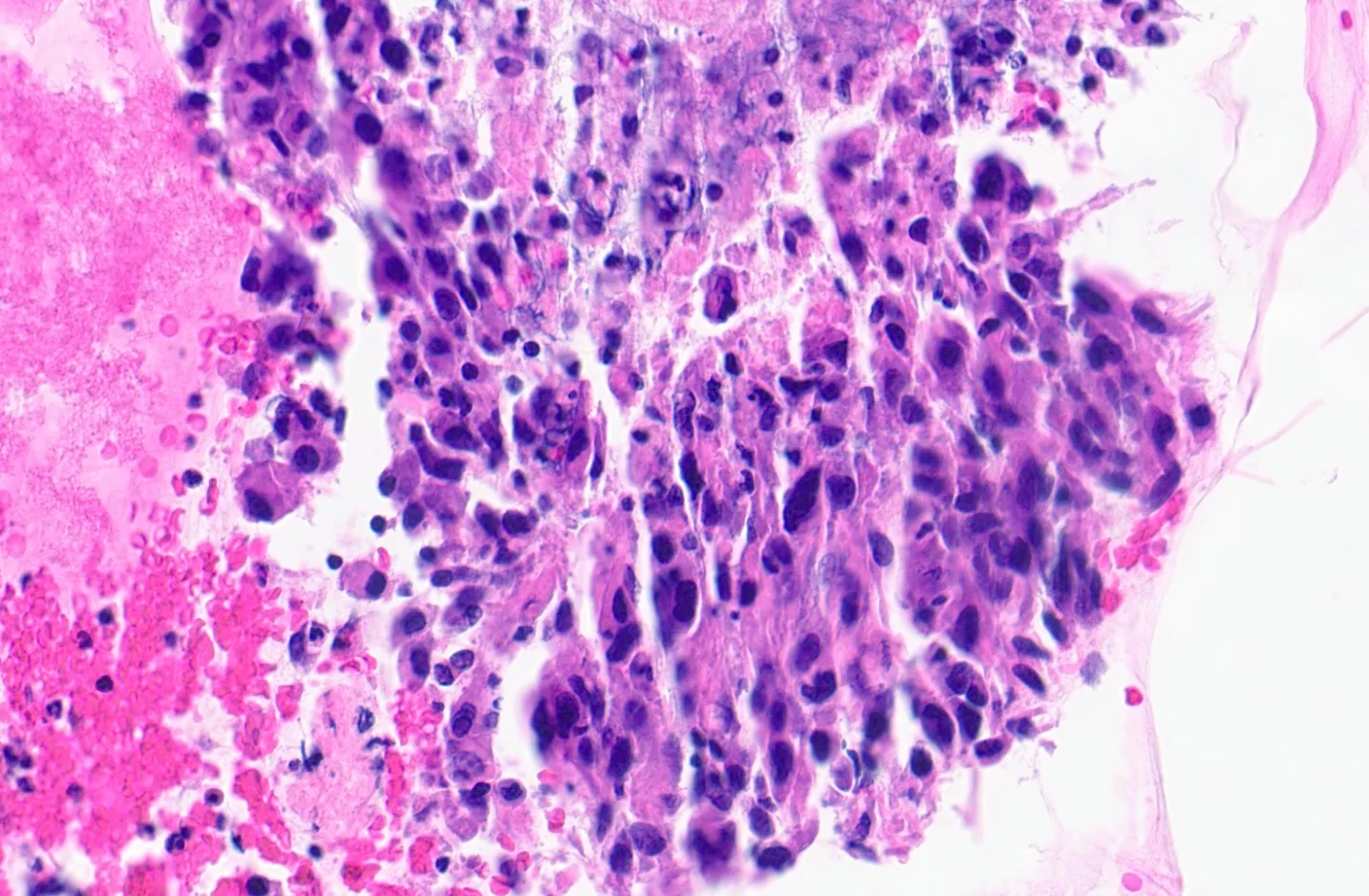
H&E. Transbronchial needle aspiration cytology cell block findings show an overtly malignant neoplasm with variably cohesive cells with hyperchromatic, pleomorphic nuclei and increased nuclear-to-cytoplasmic ratios. Scant necrosis is noted.

Examination of the pre-treatment cytopathology cell block revealed clusters of overtly malignant cells with hyperchromatic, pleomorphic nuclei, and high nuclear-to-cytoplasmic ratio, in a background of necrosis (**Figure 2**). Morphologically, the differential diagnosis included melanoma, malignant peripheral nerve sheath tumor (MPNST), lymphoma, and poorly differentiated carcinoma.

Immunohistochemical staining demonstrated strong S100 and SOX10 positivity, patchy PRAME expression, and rare weak MITF positivity. INI-1 and H3K27me3 expression were retained (wild-type pattern) (**Table 1**). The tumor cells were negative for HMB-45, Melan-A, cytokeratin AE1/AE3, SMA, desmin, MyoD1, myogenin, ERG, and CD45. Diffuse S100 and SOX10 positivity established melanocytic/neural-crest lineage; retained INI-1 and H3K27me3 argued against MPNST; and negative CD45 and AE1/AE3 excluded lymphoma and carcinoma, respectively. Because the specimen was paucicellular and necrotic, the melanocyte-specific markers HMB-45 and Melan-A were negative—a pattern that can occur in poorly differentiated or dedifferentiated melanoma. Consequently, the diagnosis was not established on morphology and conventional immunohistochemistry alone, although the overall immunophenotype supported melanoma.

**Table 1:** Immunohistochemical profile of the pre-treatment biopsy and its diagnostic interpretation.

Marker	Result	Interpretation
S100	Positive	Melanocytic/neural-crest lineage
SOX10	Positive	Melanocytic/neural-crest lineage
PRAME	Patchy positive	Supports melanoma
MITF	Rare, weak positive	Melanocytic lineage
HMB-45	Negative	May be lost in poorly differentiated melanoma
Melan-A	Negative	May be lost in poorly differentiated melanoma
INI-1 (SMARCB1)	Retained	Argues against malignant peripheral nerve sheath tumor
H3K27me3	Retained	Argues against malignant peripheral nerve sheath tumor
AE1/AE3	Negative	Argues against carcinoma
CD45	Negative	Argues against lymphoma
ERG	Negative	Argues against vascular neoplasm
SMA, desmin, MyoD1, myogenin	Negative	Argues against myogenic/smooth-muscle tumor

Molecular analysis was performed using the Northwestern Medicine Solid Tumor Next Generation Sequencing (NGS) panel (PGDx elio tissue complete, >500 genes; U.S. FDA-cleared) for DNA and the FusionPlex Solid Tumor panel (Archer FusionPlex Pan-Solid Tumor v2, 142 genes) for RNA. The tumor was microsatellite stable with a high tumor mutational burden (TMB) of 39.2 mutations per megabase. Sequencing demonstrated *NRAS* (G13R), *PTEN*, and *TERT*-promoter alterations; no *BRAF* V600 or other targetable driver mutation was identified (**Table 2**). The mutational spectrum was dominated by C>T transitions at dipyrimidine sites, consistent with a UV radiation-associated signature (COSMIC Signature 7). Taken together, the immunophenotype, the canonical melanoma driver alterations, and the UV signature were most consistent with melanoma.

**Table 2:** Molecular profile of the pre-treatment biopsy (DNA and RNA next-generation sequencing).

Feature	Result
Tumor mutational burden	High — 39.2 mutations/Mb (assay threshold ≥16)
Microsatellite status	Stable
*NRAS*	p.G13R (c.37G>C)
*TERT* promoter	c.-124C>T
*PTEN*	p.P169L (c.506C>T)
*TP53*	p.R213* (c.637C>T)
*NOTCH3*	p.R558C (c.1672C>T)
Copy-number alterations	None detected
Gene fusions/rearrangements	None detected (142-gene RNA fusion panel)
Pertinent negatives	*BRAF* (including V600) and *KIT* wild-type; *ALK*/*RET*/*ROS1*/*NTRK1–3* fusions negative

Clinical examination and imaging revealed no cutaneous, mucosal, or ocular primary and no additional sites of disease. Although UV signatures suggest a cutaneous origin, they may be seen in melanomas of unknown primary, as supported by the absence of cutaneous findings in our patient. With no identifiable primary lesion, the case was thus interpreted as *de novo* stage IV melanoma of unknown primary presenting as a solitary pulmonary mass.

Given the resectable but bulky oligometastatic disease, as well as the extent of pulmonary artery involvement and high morbidity anticipated with upfront pneumonectomy, the multidisciplinary team elected a neoadjuvant-adjuvant strategy informed by SWOG S1801 (Patel et al.) (2). Pembrolizumab 200 mg was administered intravenously every 3 weeks for three cycles before surgery and was planned for 15 cycles after resection, regardless of pathologic response, for a total of 18 perioperative cycles. Given her SLE and potential increased risk of grade 3–4 immune-related adverse events (irAEs), single-agent therapy was selected over combined neoadjuvant CTLA-4 and PD-1 checkpoint inhibition. Because human lactation data for pembrolizumab are lacking, the manufacturer advises against breastfeeding during treatment and for 4 months after the final dose, and the patient was counseled accordingly.

**Figure 3:**
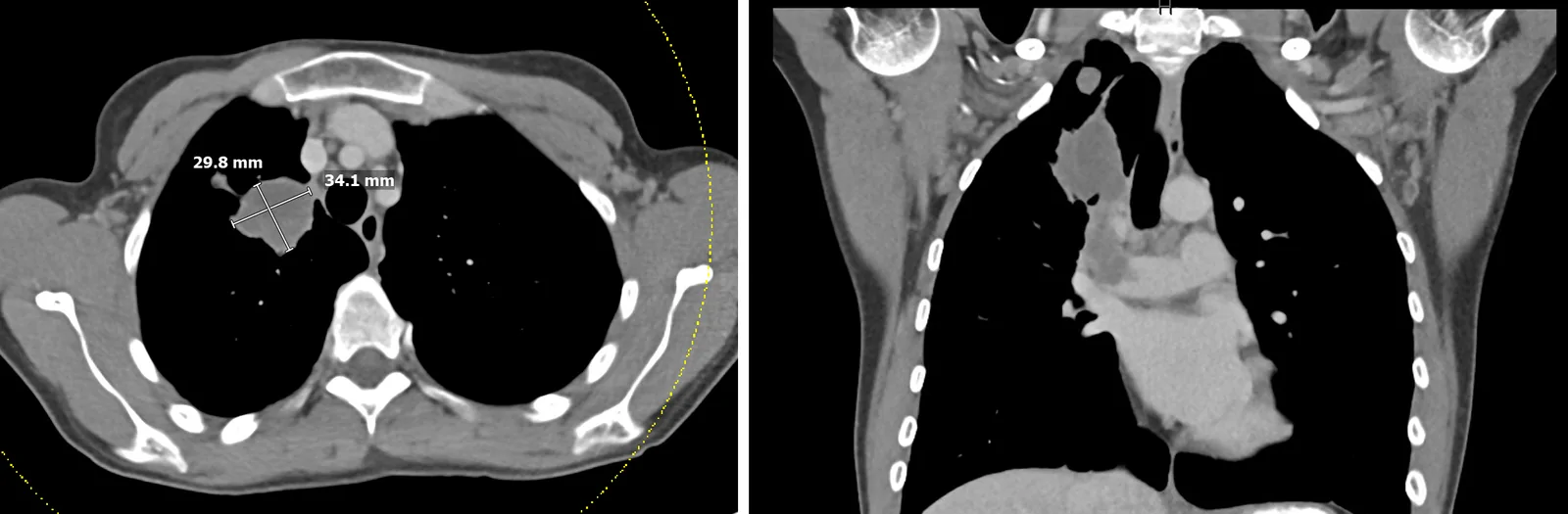
CT performed after three cycles of neoadjuvant pembrolizumab, two days prior to pneumonectomy and pulmonary artery reconstruction.

Pembrolizumab was well tolerated; the only notable toxicity was a postpartum SLE flare managed with hydroxychloroquine and low-dose prednisone, co-managed by Rheumatology. No grade 3–4 irAEs were observed. A PET-CT obtained 90 days after initiation of neoadjuvant pembrolizumab demonstrated a residual 3.4-cm mass with decreased occlusion of the right pulmonary artery (**Figure 3**).

**Figure 4:**
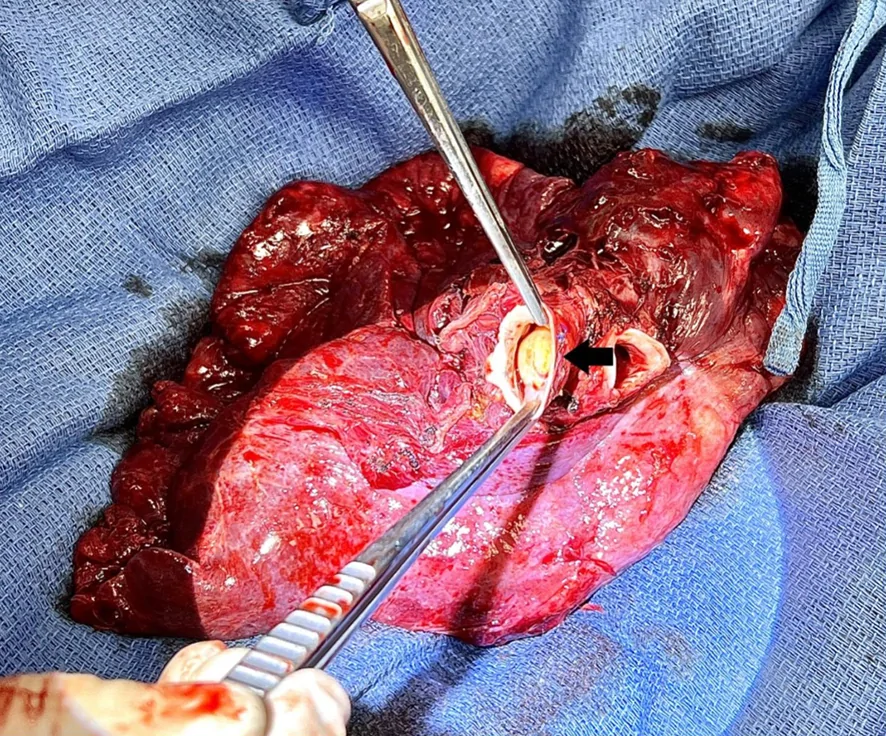
Right pneumonectomy. *Black arrow* indicates tumor within the right main pulmonary artery.

After completion of neoadjuvant treatment, the patient underwent sternotomy, right pneumonectomy, and right pulmonary artery resection and reconstruction on cardiopulmonary bypass (**Figure 4**). Intraoperatively, the tumor was found to involve the superior segmental artery, necessitating pneumonectomy. The bronchial stump was buttressed with an intercostal muscle flap. The patient tolerated the procedure well and was discharged on postoperative day 5, with another mild SLE flare managed conservatively. On final pathology, all sections of the tumor bed showed necrosis, fibrosis, and aggregates of histiocytes with no viable tumor, consistent with >99% treatment effect. Applying the International Neoadjuvant Melanoma Consortium (INMC) criteria, in which pathologic complete response (pCR) is defined as 0% residual viable tumor, the findings represented a pCR (19). Surgical margins were negative for malignancy, and all sampled lymph nodes—two subcarinal and five hilar—were benign.

She began adjuvant pembrolizumab on postoperative day 41 and completed all 15 planned adjuvant cycles per the SWOG S1801 protocol (2). Surveillance imaging obtained following completion of adjuvant therapy has shown no evidence of disease, and the patient remains asymptomatic at 21 months after surgery.

## Discussion

**Figure 5:**
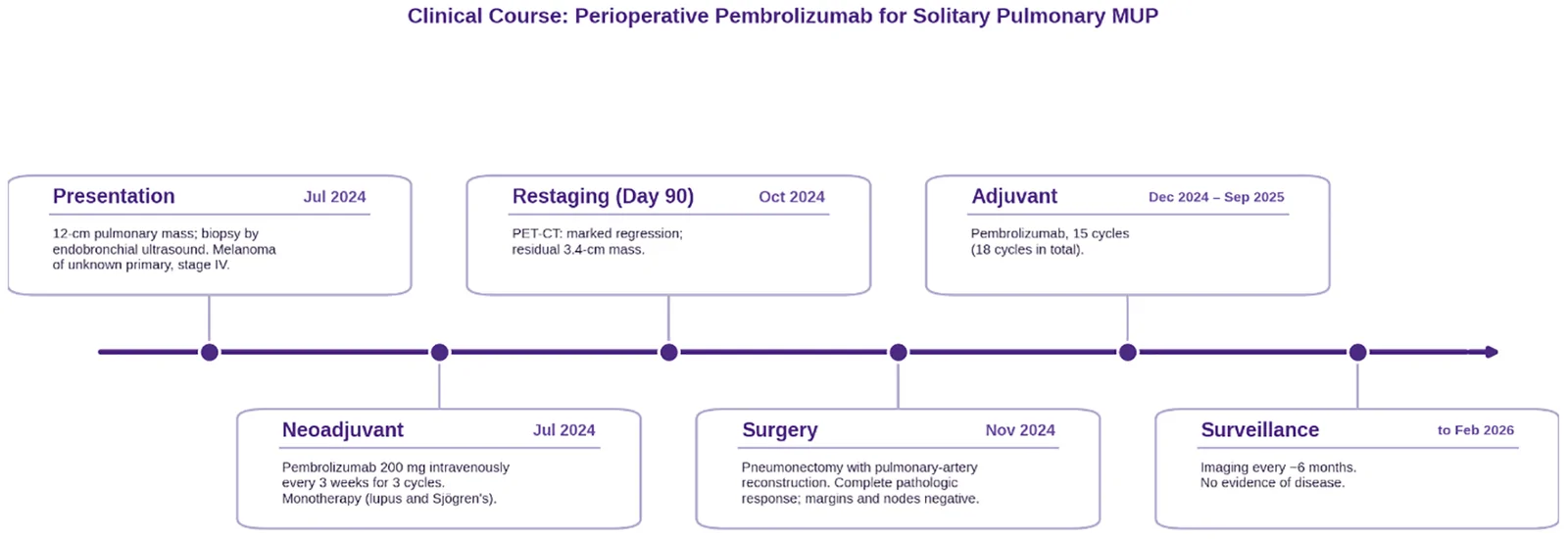
Timeline of the clinical course, from presentation and diagnosis through neoadjuvant-adjuvant pembrolizumab, surgery, and surveillance.

This case illustrates an exceptional pathologic response to perioperative PD-1 blockade in a young peripartum woman with an isolated pulmonary metastasis from melanoma of unknown primary. Several aspects merit discussion: the biology of MUP, the rationale for a perioperative immunotherapy strategy in oligometastatic disease, the interpretation of immunogenomic biomarkers, and the use of immune checkpoint inhibition in patients with autoimmune disease and in the peripartum period. The overall clinical course, from presentation through surveillance, is summarized in **Figure 5**.

### Melanoma of unknown primary

MUP accounts for approximately 2-5% of melanoma cases and is thought to arise, in many instances, from immune-mediated regression of an occult cutaneous primary (6–8). A melanoma presenting as a solitary pulmonary lesion therefore raises a specific diagnostic question: whether it represents a metastasis from an occult or regressed cutaneous primary, or a true primary pulmonary melanoma. Primary pulmonary melanoma is exceedingly rare and remains a diagnosis of exclusion, requiring a solitary lesion with no current or prior identifiable primary (14, 16). Increasingly, however, lesions once regarded as primary pulmonary melanoma are recognized as metastases from occult or regressed cutaneous primaries on the basis of molecular profiling; in particular, a UV mutational signature argues for a cutaneous origin, because a tumor arising primarily within the lung would not be expected to carry this UV-damage-associated fingerprint (15).

In our patient, no cutaneous, ocular, or mucosal primary was identified on examination or imaging, and the tumor carried a dominant UV signature together with canonical cutaneous-melanoma drivers (*NRAS* G13R, *TERT*-promoter). These features favored a regressed or occult cutaneous origin over a primary pulmonary melanoma, and the case was best classified as MUP presenting as an isolated pulmonary metastasis. Definitive re-characterization was precluded by the complete pathologic response, but the balance of morphologic, immunophenotypic, and molecular evidence supported this interpretation. The immune-mediated regression hypothesized to underlie MUP implies pre-existing antitumor immunity, which may further prime such tumors for durable responses to checkpoint blockade (9–12).

### Perioperative immunotherapy and oligometastatic disease

Systemic therapy for this tumor was effectively limited to immunotherapy. Although the tumor carried recognized driver alterations, none was directly targetable: *BRAF*/MEK-directed therapy requires a *BRAF* V600 mutation, which was absent, and there is no approved first-line targeted therapy for *NRAS*-mutant melanoma (20). Immune checkpoint inhibition is therefore the established first-line systemic approach in advanced and perioperative melanoma regardless of driver status. Randomized data support neoadjuvant–adjuvant immunotherapy in resectable melanoma: SWOG S1801 demonstrated superior event-free survival with perioperative pembrolizumab compared with adjuvant-only pembrolizumab (2), and NADINA showed high pathologic response rates with neoadjuvant ipilimumab plus nivolumab (3).

These trials, however, enrolled predominantly stage II–III resectable melanoma, and neither specifically addressed oligometastatic stage IV disease or MUP; extrapolation to the present setting should therefore be cautious, and prospective evaluation in this subgroup is needed before routine adoption. The regimen used here mirrors S1801—pembrolizumab 200 mg intravenously every 3 weeks for three neoadjuvant cycles, surgery, and then 15 adjuvant cycles, for approximately one year of perioperative therapy. Although our patient had stage IV disease by virtue of a pulmonary metastasis, her disease was oligometastatic and technically resectable, and a perioperative, curative-intent strategy was pursued.

Neoadjuvant therapy offered two advantages in this setting: cytoreduction to facilitate a high-risk pneumonectomy with pulmonary-artery reconstruction, and *in vivo* assessment of tumor sensitivity to inform subsequent therapy. Patients most likely to benefit from this approach are those with resectable, clinically detectable disease, adequate performance status, and no contraindication to immunotherapy; the choice between combination and single-agent checkpoint blockade must weigh the greater efficacy of dual blockade against its substantially higher toxicity, particularly in patients with autoimmune disease. In our patient, single-agent pembrolizumab was selected on this basis and produced a pCR.

### Immunogenomic biomarkers: UV signature, TMB, and ctDNA

The tumor’s immunogenomic profile offers a plausible biologic explanation for the depth of response, but these features should be interpreted with caution. TMB, which is a quantitative count of somatic mutations per megabase, correlates with neoantigen load and, in metastatic melanoma, with the likelihood of response to PD-1 blockade (21, 22). It is important to distinguish TMB from the UV mutational signature: whereas TMB measures the quantity of mutations, a mutational signature describes their qualitative pattern and underlying etiology. The UV signature (characterized by C>T transitions at dipyrimidine sites; COSMIC Signature 7) reflects the specific mutational process of UV-induced DNA damage and serves as a molecular fingerprint of cutaneous origin, whereas a high TMB may arise from UV exposure or from other mutagenic processes. The two are related but not equivalent, and both were present in this tumor. Importantly, although high TMB is associated with immunogenicity, it is not an established or validated biomarker for selecting perioperative therapy in melanoma and is not used to guide neoadjuvant treatment decisions in current practice; the association observed here is therefore hypothesis-generating rather than outright predictive.

Circulating tumor DNA (ctDNA) was not assessed in this case. ctDNA has an emerging role in melanoma for early response assessment, detection of molecular residual disease, and surveillance, and its incorporation might have provided a noninvasive complement to imaging in monitoring this patient (23). Given accumulating evidence that perioperative ctDNA can track response and detect molecular residual disease earlier than imaging, we recommend prospective ctDNA collection in future perioperative melanoma protocols to complement pathologic and radiologic assessment.

### Immune checkpoint inhibition in autoimmune disease and the peripartum setting

Patients with pre-existing autoimmune disease have historically been excluded from immunotherapy trials, and the available data are largely retrospective. In a multicenter series of patients with advanced melanoma and pre-existing autoimmune disorders treated with anti-PD-1, disease flares occurred in roughly one third of patients but were usually mild and manageable, discontinuation was infrequent, and antitumor efficacy was preserved; rheumatologic conditions such as SLE were among the most likely to flare (24). These observations are consistent with our patient’s course: she experienced manageable SLE flares controlled with hydroxychloroquine and low-dose prednisone, without grade 3–4 irAEs, and achieved a pCR. This case adds to the evidence that anti-PD-1 monotherapy can be delivered with careful multidisciplinary monitoring in selected patients with rheumatologic disease, though such decisions must remain individualized.

The peripartum context introduced additional considerations. Although pembrolizumab’s large size suggests minimal milk transfer and likely degradation in the infant gastrointestinal tract, the data on pembrolizumab excretion in breast milk are lacking. Consequently, the manufacturer recommends against breastfeeding during treatment and for up to 4 months after the final dose, and the patient was counseled accordingly (25). Immune checkpoint inhibitors are also generally avoided during pregnancy given the central role of the PD-1/PD-L1 axis in maintaining maternal-fetal tolerance (26); accordingly, systemic therapy was initiated only after delivery.

### Limitations

This report has important limitations. It describes a single patient, and the observed association between the tumor’s immunogenomic features and its exceptional response is biologically plausible but hypothesis-generating and cannot establish causality or predictive value. Molecular characterization was constrained by scant, necrotic biopsy material, and definitive pathologic re-characterization was precluded by the complete response. Longer follow-up and the aggregation of similar cases with integrated molecular and pathologic data will be necessary to determine whether specific immunogenomic profiles can reliably stratify patients for perioperative immunotherapy.

## Conclusion

To our knowledge, this case is among the first detailed descriptions of perioperative PD-1 blockade producing pCR in a peripartum patient with oligometastatic MUP characterized by UV-signature mutagenesis and high TMB. While these features may help explain heightened immune sensitivity, conclusions must be cautious. Integrated molecular profiling and standardized pathologic response assessment should be incorporated into future perioperative melanoma studies to clarify predictive value and optimize patient selection.

## Data Availability

The original contributions presented in the study are included in the article/supplementary material. Further inquiries can be directed to the corresponding author.
